# International Committee on Taxonomy of Viruses and the 3,142 unassigned species

**DOI:** 10.1186/1743-422X-2-64

**Published:** 2005-08-16

**Authors:** CM Fauquet, D Fargette

**Affiliations:** 1 International Laboratory for Tropical Agricultural Biotechnology, Danforth Plant Science Center, 975 N. Warson Rd., St. Louis, MO 63132 USA; 2 Institut de Recherche pour le Developpement, BP 64501, 34394 Montpellier cedex 5, France

## Abstract

In 2005, ICTV (International Committee on Taxonomy of Viruses), the official body of the Virology Division of the International Union of Microbiological Societies responsible for naming and classifying viruses, will publish its latest report, the state of the art in virus nomenclature and taxonomy. The book lists more than 6,000 viruses classified in 1,950 species and in more than 391 different higher taxa. However, GenBank contains a staggering additional 3,142 "species" unaccounted for by the ICTV report. This paper reviews the reasons for such a situation and suggests what might be done in the near future to remedy this problem, particularly in light of the potential for a ten-fold increase in virus sequencing in the coming years that would generate many unclassified viruses. A number of changes could be made both at ICTV and GenBank to better handle virus taxonomy and classification in the future.

## Introduction

The VIIIth ICTV Report [1] lists more than 6,000 viruses classified in 1,950 species, themselves classified in 391 taxa. Thus, it was surprising to discover that GenBank (part of the International Nucleotide Sequence Database Collaboration, comprising the DNA DataBank of Japan, the European Molecular Biology Laboratory, and GenBank, which is located at the National Center for Biotechnology Information (NCBI) in the U.S. National Institutes of Health) has collected sequences belonging to 3,142 "species" of viruses not present in the ICTV current master list of 2005.

For the past 20 years, GenBank has increased the number of sequences stored from one in 1982 to 42 million in 2002, and has increased the number of tools at the disposal of users to the point where today it is an absolutely necessary system for molecular biologists and virologists world-wide. In particular, GenBank has developed a complete database for taxonomy, including virus taxonomy, allowing virologists to search all viruses and to identify new sequences and new virus names. Were it not for the existence of this excellent system, we would not have known of the existence of these 3,142 unassigned "species" of viruses.

ICTV also has been very active in the last 20 years, incrementally increasing the number of taxa and virus names from 369 in 1985 to 7,881 in 2004. Not only have the numbers increased exponentially (about 20-fold), but the complexity of virus nomenclature and taxonomy has become tremendously complicated and controversial, even puzzling for some. However, the overall stability of this virus classification, established in 1962 [2], is quite remarkable in that, for example, names of all genera and families established in the 1980s are still in use in 2005. The advancement with the most impact was the definition of a virus species [3], which still is not fully understood by most virologists. Therefore, it is not surprising that its application in terms of names and concept causes problems such as the one discussed in this paper.

The listing by GenBank, but not by ICTV, of 3,142 unassigned "species" clearly demonstrates a general problem of the application of the definition of virus species and that ICTV and GenBank must work in concert to cope with the day-to-day reality of virology and virus classification, and that collectively we need to improve the system in such a way that we will rapidly classify so-called "species" of viruses and change the current system, so that we do not find ourselves in a similar situation in the future.

This paper will review the probable causes of the present situation and will identify actions that could remedy the situation both at GenBank and at ICTV. Finally a brief description of the Taxonomic Proposals Management System (TPMS), a new database to electronically handle all taxonomic proposals for ICTV, will be provided.

## Discussion

### What are the causes of such a situation?

#### Review of the species concept

A virus species, as with all other taxonomic levels, is a concept devised by humans to describe concrete (real) biological viral entities. The definition of a virus "species", established [4] and accepted by ICTV [3,5], is as follows: "A virus species is a polythetic class of viruses that constitutes a replicating lineage and occupies a particular ecological niche". This definition is broad enough to allow considerable flexibility. It is the responsibility of the virologists interested in each virus family to apply this definition to their favorite viruses and to decide how many and which species should comprise a particular genus of a particular family [6]. Typically, this is the work of ICTV study-groups, whose members are experts in particular virus families [7]. Virus sequences are physical realities that are not species but entities assigned to a particular species [8]. For example, the 24 bluetongue viruses are assigned to the species *Bluetongue virus *in the genus *Orbivirus*, family *Reoviridae*; thus, 24 viruses assigned to a single species. Therefore sequences entered in GenBank must be associated with the name of a virus isolate. The term virus isolate has no specific definition and can be applied to any virus, so long as that virus has existed for some time. Several terms are used by virologists to define a virus entity below the species level and are somewhat variable and confusing, but for virus sequences the term "virus isolate" seems the most appropriate, practical and useful [9].

#### Sequence and information collection

The intent of GenBank is to collect sequences but the author, the person who enters the sequences in the database, is responsible for the accuracy of the sequences as well as for the validity of the associated information about the sequences, which includes the taxonomic information about the virus in question. The primary taxonomic information pertains to an isolate of a previously recognized virus or of a newly recognized virus. Consequently, if the author errs in naming the virus or in its classification, the entry will be incorrect and might remain incorrect for a long period of time. GenBank has personnel qualified to review all entries and they effectively and immediately correct a large number of obvious mistakes. However, with regard to "new species", GenBank personnel do not have the means to determine whether this new taxon is justified and acceptable or not. Obviously, this policy of GenBank cannot be changed from without, but it is certainly possible for GenBank to slightly modify the Entrez form in order to build in safety nets to avoid such situations in the future.

#### GenBank Entrez form and software

The Entrez form of GenBank is a general form for all organisms, and one of the first questions asks the name of the organism. Whereas viruses are not organisms, this question immediately causes ambiguity because it is not clear whether the isolate name or the name of the species to which it belongs should be indicated, notwithstanding the fact that many investigators have not yet understood the difference between an isolate name, corresponding to a specific sequence of a virus, and the conceptual species name, to which a particular isolate has been assigned [10,8] (see above). The second drawback comes from the fact that the software dealing with Entrez and connected to the Taxonomy database of GenBank will by default assign any new virus name in the field "Organism" to a new "species" if it does not exist in the ICTV Master list, from which the current valid classification is derived. This implies that most of the 3,142 "species" are in fact virus isolates only (concrete entities), rather than species (conceptual). The third problem arises from the fact that many virus species names and virus names are identical in the words that compose them, except that species names are written in italics and are not abbreviated, while virus names are not italicized and may be abbreviated [10-13]; unfortunately, databases can only use ASCII characters and cannot accept italics. It is particularly unfortunate that we are in this situation and that different names have not been created for virus species, but this is a reality that cannot be changed, at least in the short term, and consequently we need to find a way to finesse these problems and still adhere to acceptable virus nomenclature. It seems that the only possibility is to redesign software to show taxonomy as it should be shown, and to restore the difference between the two names, if not by italics at least by some equivalent means. The fourth problem also is associated with the ASCII code and that is the impossibility of using Greek letters, which are incorporated in many phage names. For this, one solution might be to have ICTV change these names slightly by spelling out the Greek letters, c.f., "Enterobacteria phage lambda", instead of "Enterobacteria phage λ".

#### Exponential increase in sequencing

**Figure 1:**
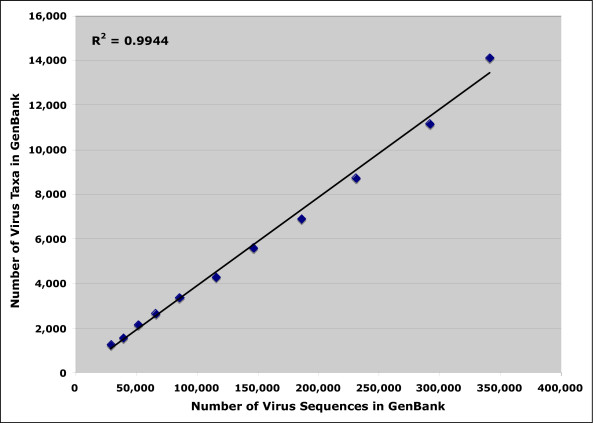
Linear correlation between the number of virus sequences recorded at GenBank between 1996 and 2005 and the number of virus taxa and virus isolates.

Because of lower costs and more efficient technology, sequencing is increasing exponentially. Known viral sequences already are in the hundreds of thousands and it is conceivable that millions more will become available in the coming years. The number of virus taxa and isolates also increases exponentially and is therefore linearly related to sequencing activities, as shown in Figure 1. It is therefore critical to have all data stored and named properly and with taxonomic updates in real time.

#### Taxonomic knowledge and consciousness of virologists

Not all virologists determining sequences are fully aware of or interested in virus taxonomy. These people are the primary source of information and they consequently are the primary sources of data entry errors. Left adrift, they will not make efforts to solve these particular sorts of problems, so other solutions should be put in place to remedy the problem. Insisting that they follow taxonomic rules seems incompatible with the general "author" principle of GenBank, but it is possible to "invite" them to enter the proper information directly on the Entrez form.

#### ICTV organization

**Figure 2:**
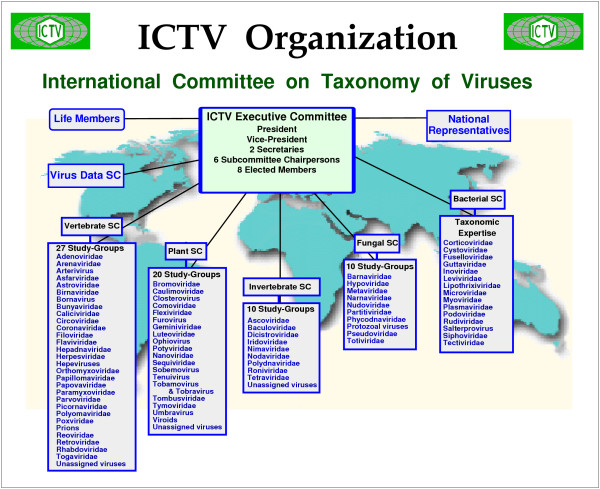
Diagrammatic organization of the ICTV.

The organization structure of ICTV, the generally accepted leader in viral taxonomy, can be conceived of as pyramidal [7]. The base of the pyramid comprises more than 500 virologists in six Subcommittees, divided into 82 specialty study-groups (SG), each representing a family of viruses or an unassigned genus (ICTVnet: ) (Fig. 2).

Each SG comprises from three to 15 experts selected for their expertise in recognized and new viruses and new sequences of viruses in each family; obviously this is an increasingly difficult task, given the rapid increase in available data. These 82 SGs present taxonomic proposals from the level of species to the level of order, the proposals are reviewed by the Executive Committee (EC) of the ICTV and, 18 months later, through a posting on ICTVnet and two rounds of discussions, are then ratified (or not) and voted upon (or not) by the ICTV membership at large. This system, although well organized and in full compliance with the international code for virus taxonomy and nomenclature [14], is neither adapted to a large number of new isolates and species nor to a quick acceptance, so that changes are envisioned. In addition, by statute, the ICTV does not have the mandate to deal with virus isolates, which are not taxa. This is a fundamental problem because no organization is responsible for certifying isolate names or for determining to which virus species they belong. However, ICTV, for historical reasons, lists a large number of unclassified virus isolates and SG members are the most knowledgeable people to deal with such an issue. Obviously, this should be considered by ICTV in order to solve this issue of unassigned viruses erroneously purported to be "species" and found in various databases.

### What are the solutions?

It is not the responsibility of the authors to decide whether their new virus isolates belong to an existing or a new species, not the responsibility of GenBank to police authors and organize virus taxonomy and nomenclature, and not the responsibility of ICTV to deal with non-taxonomic issues, such as isolate names, but it is our communal responsibility to work together to make the system functional; and it certainly is feasible. Here we call for collaboration of all parties to establish a generally satisfactory system for virus taxonomy and nomenclature of virus isolates, sequence data, and all other virological data. Obtaining solutions will necessitate the collaboration of all those involved in producing, collecting and classifying viruses.

#### Classifying the 3,142 unassigned virus names in GenBank

Placing the 3,142 unassigned "species" (which likely are simply "viruses") registered at GenBank will require manual classification by the ICTV SG members. Most of these "species" are probably isolates of recognized species and are in the GenBank database by default as new "species" because, whether they have been named or not, they have not been entered in the GenBank forms. Some of these GenBank "species", however, may actually be new species that have not yet been submitted to ICTV and, if so, they should be proposed through the normal channels of the Taxonomic Proposals (TaxoProps) for evaluation and official ratification before they can be recognized as new species.

#### Improving the management of taxonomic proposals

As a remedy to the general problem of virus classification, we suggest a two-tier plan. Firstly, GenBank would work with ICTV to provide to the authors rolling menus, or automatic writing, offering a list of all the official ICTV names to select from. This would help avoid spelling errors or the creation of incorrect new names by authors. In the case of an unaccepted (or provisional) virus name, the default should be "unassigned virus in the genus, or in the family, or in the order or in the virus kingdom". This assignation to an "unassigned category" would trigger an email to the relevant ICTV SG chair and to some EC members so that ICTV will be immediately aware of this new entry. This information also would be stored in a proposed new database called TaxoProp Management System (TPMS), with a pending status to be resolved promptly (see below). The second tier of the remedy would be to establish the TPMS database to handle all new TaxoProps containing information originating from an SG member, from any individual virologist or directly from GenBank.

#### Changing ICTV to work in real time

Resolving the mechanics of the system is only a partial solution and more details are needed in order for this to be functional in real time. ICTV has established a sophisticated and democratic system to receive, review, discuss, re-discuss and finally approve taxonomic proposals, whether they concern a new species or a new order [15]. This system is very sophisticated in principle but is too complex and takes too long to be compatible with modern virology work. There are, however, fundamental and practical differences between a TaxoProp for a new species and a TaxoProp for everything else, and that is that species are solely determined by specialty groups (ICTV SGs) and rarely by the public or the ICTV EC members. The only envisioned ICTV concern would be to make certain that the virus species name follows the ICTV International Rules [14]. It would therefore be possible to computerize the verification of the names in order to "bypass" ICTV EC scrutiny and only then obtain approval of the ICTV membership by electronic voting. This in turn would considerably reduce the turn-around time: essentially, as soon as a name would be proposed by an ICTV SG, it would be automatically verified and accepted if all the rules were followed.

#### Increasing relationships between ICTV and GenBank

In order to have taxonomic consistency, it is absolutely essential that we strengthen the relationships between ICTV and GenBank. This could be done with an electronic link between TPMS and GenBank, supplemented with improved and regular personal relations. For example, GenBank has appointed virus experts for a number of families of viruses. It would be more efficient to have GenBank somehow linked directly to the SG chairs of ICTV, so that GenBank would get taxonomic experts for advice in virus taxonomy. This, in turn, would provide a more active role for the SG members and would activate the overall system of virus taxonomy.

### The TPMS database?

**Figure 3:**
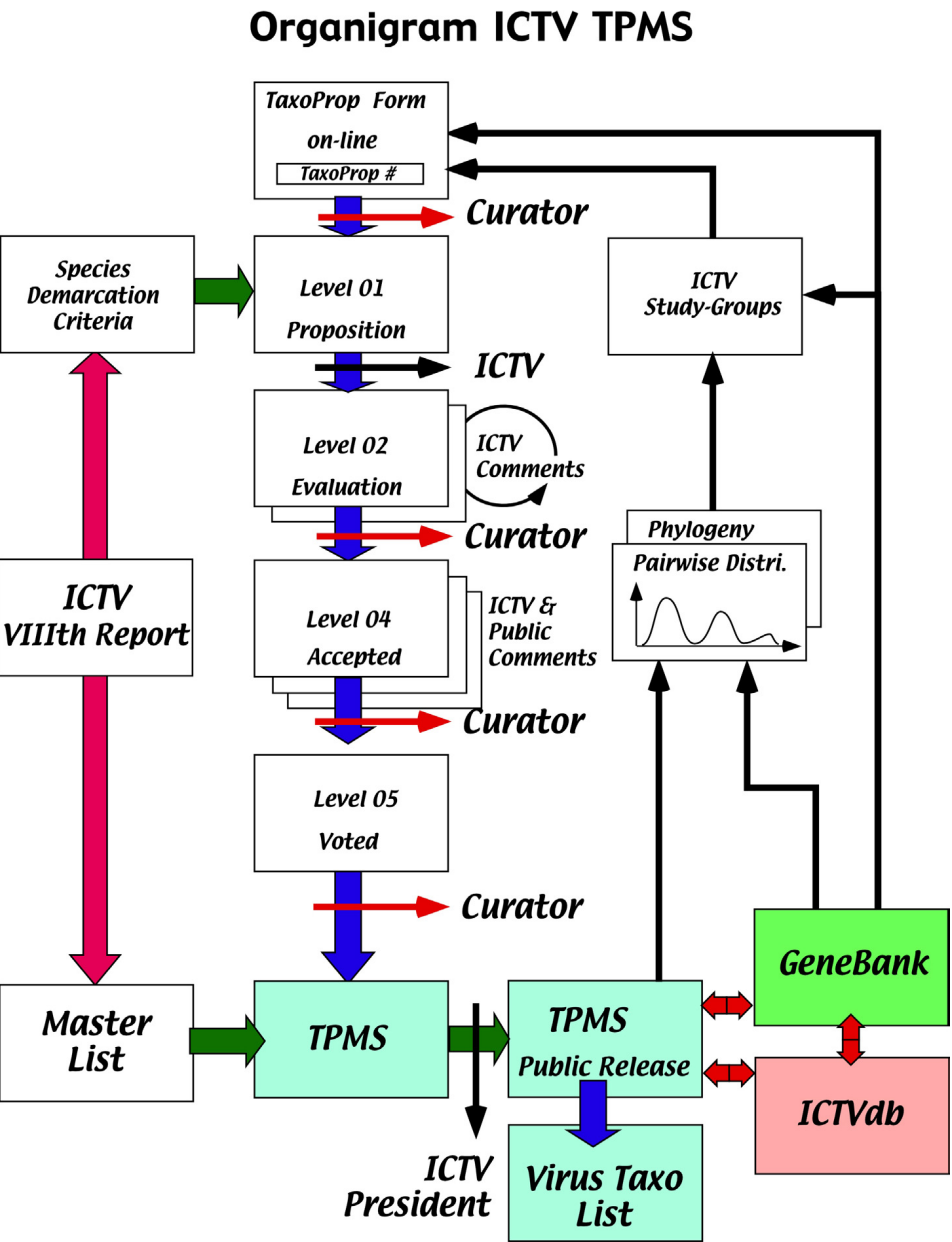
Diagrammatic organization of the TaxoProp Management System (TPMS) database.

Due to the rapid increase in the number of TaxoProps and the increasing difficulty in manually handling several thousand taxonomic names, it is planned by ICTV to create a database to handle all ICTV taxa, from inception to acceptance, and from species to order (Fig. 3).

#### Overall description of the TPMS

The goal of the TPMS database is to electronically handle all the virus taxa at large (including virus isolates), i.e., those that have been inherited from previous reports, those that have been approved between 1999 and 2005, those in the process of being approved and those that will be approved in the future.

At any time it will then be possible to:

1- certify the approval status of any TaxoProp

2- elaborate a complete list of approved taxa

3- establish an electronic link with GenBank and all other virus databases

#### Structure of the TPMS database

The database will be accessible on line to all ICTV members and to the public at large. The database will comprise a collection of individual files, each one corresponding to a single taxon (order, family, subfamily, genus, and species), and in addition will record isolates. Each TaxoProp will progress from the initial proposal to the approval of the taxon by the ICTV. The bulk of the database will be constituted by all the approved taxa and individual viruses and should be exportable to other databases for future use.

#### Approval process of the TaxoProps

**Figure 4:**
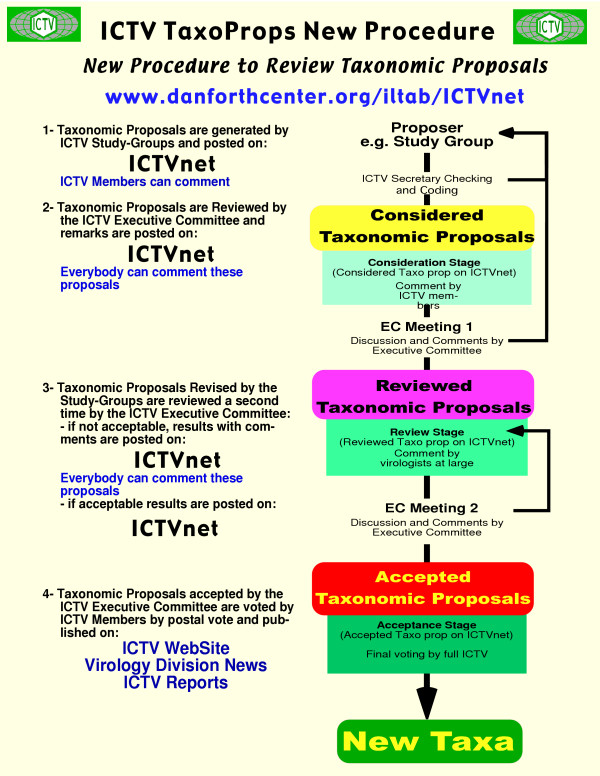
Diagrammatic organization of the taxonomic proposals system at ICTV.

The TaxoProp approval system of ICTV is depicted in Figure 4. When a TaxoProp form is filed on line, it is then considered by ICTV and becomes "Considered". A TaxoProp will consist of the proposal itself, of arguments provided to support the TaxoProp, and by attached documents to be downloaded by the reader. A unique TaxoProp number will be automatically generated when a form is completed or when a TaxoProp is modified by the author. After consideration, it becomes "Considered", and comments of the ICTV are added on line and available to the public at large. Comments can be added by any virologist and the author of the TaxoProp can respond on line. The change of level will be done through a voting system in which ICTV EC members can view the votes at any time and will be operated by the TPMS curator. When accepted by ICTV, the TaxoProp becomes "Accepted" and comments are no longer added. When the ICTV at large has voted to approve the TaxoProp, it becomes "Voted" and is *de facto *a ratified taxon of the ICTV.

#### Filing TaxoProps

TaxoProps can be filed by any virologist, but it is likely that most will be filed by SG members. There will be a set of TaxoProps to choose from on line according to the sort of taxon proposed: an isolate (although not a formal taxon), a species, a type species, a genus, a subfamily, a family, an order, or any other taxon proposed by any virologist. Higher taxa cannot be proposed unless a lower level has been proposed and accepted. A genus must have a type species and at least one species or a list of species in the genus. Any proposed new taxa must have a name following the ICTV Code [14].

#### Link with species demarcation criteria lists

For each genus, it will be possible to list the species demarcation criteria of that genus for the benefit of the author and readers of the species TaxoProps. These lists will be extracted from the VIIIth Report and from subsequent updates. It is envisioned that these lists of species demarcation criteria will become useful tools for ICTV to search for a better means of making the overall virus taxonomy more uniform in the future.

#### Special case of TaxoProp isolates

Although isolates do not comprise formal taxa, recording and listing them are indispensable for the creation of species taxa and also for practical purposes. Virus species being defined from properties of virus isolates, ICTV records the existence of isolates in order to define species. There are no formal rules for naming isolates and, consequently, any name could potentially be accepted. However, it is common sense that these names follow previously established names and follow somewhat virus species names rules to avoid complete chaos [14,9].

#### Accepting TaxoProp species

TaxoProp species, after being proposed by the SGs, will automatically be reviewed to ensure that they follow the ICTV International Code and, if they do, will automatically be approved electronically. However, a vote by the ICTV membership at large will still be necessary for it to be considered as ratified by the ICTV; this also could be done electronically.

#### Approved taxa

All approved taxa, because they have been inherited, will be entered in the TPMS, so that the database will be updated automatically and in real time and can be linked to GenBank, ICTVdb (ICTV database) and other databases. Files for each taxon will archive all the comments and votes so that we can always return to this information if needed. However, public access to the TPMS for the ratified ICTV Master List will remain under control of the President of ICTV.

#### Generating the ratified list of taxa

Using TPMS, it will be possible to generate a complete list of approved taxa at any time, and this will be possible either alphabetically or taxonomically. Accepted taxa also will be presented according to the order of presentation of virus taxonomy: dsDNA, ssDNA, rtDNA, rtRNA, dsRNA, ssNRNA, ssPRNA, SAT (Satellites), VIR (Viroids), UN (unassigned). Within each category, the families will be classified according to the Order of Presentation of the Virus. Taxonomy Species will be assigned to a genus and classified alphabetically within the genus. The species taxa should comprise at least one isolate but include as many as described.

#### Searching the TPMS database

Any virologist will have access to the TPMS database on line anytime so that he or she can verify names, either with an alphabetical pull down menu system or by use of a search engine.

#### Linking the TPMS database to other databases

TPMS will *de facto *become the definitive reference for virus taxonomy and nomenclature. Once approved by the ICTV President, typically after each ICTV EC meeting or after a certain period of time (such as six months), the TPMS database will be electronically linked with GenBank, ICTVdb, and other associated databases to ensure complete homogeneity of the virus taxonomy and nomenclature. This tool will be particularly important for the members of the International Virus Database Network, who recognize the critical need for such a system, and to homogenize and update in real time the virus taxonomy and nomenclature.

## Conclusion

Because sequencing is much less expensive and increasingly done these days, it is assumed that investigators will generate many more sequences of known and unknown viruses in the very near future. The number of sequences is increasing exponentially and so will the number of virus names and virus taxa, as they are directly correlated with sequencing activities. It is clear that past management of virus taxonomy will become inadequate commensurate with this increase and that more rapid and better-adapted management is required. The electronic connection between databases storing sequences, storing biological data or handling taxonomic proposals is the only solution for the future. Human intervention is indispensable, but should be restricted to the decision-making process; all other interventions might best be done electronically. This will mean better collaboration between the people responsible for the different types of databases, the people involved in the ICTV organization, and individual investigators themselves. Software will have to be adapted or devised to better fit virus taxonomy and nomenclature, ICTV will have to better appreciate day-to-day realities such as dealing with virus isolates, and scientists will have to be better educated in virus taxonomy. Regarding the decision-making process for virus species and isolates, a much greater role for the ICTV SGs will be necessary because the ultimate expertise resides with them in determining which new viruses fit into existing taxa and which must be placed in defined new taxa. Consequently, it would be best for ICTV to work more closely with GenBank to establish the electronic TPMS database to work in real time and to cope with an acutely burgeoning number of taxa.

## Competing interests

The author(s) declare that they have no competing interests.

## Authors' contributions

The authors contributed equally to the writing of this paper.
